# Within-Stand Distribution of Tree Mortality Caused by Mountain Pine Beetle, *Dendroctonus ponderosae* Hopkins

**DOI:** 10.3390/insects11020112

**Published:** 2020-02-10

**Authors:** José F. Negrón

**Affiliations:** USDA Forest Service, Rocky Mountain Research Station, 240 West Prospect, Fort Collins, CO 80526, USA; jose.negron@usda.gov

**Keywords:** *Pinus ponderosa*, bark beetles, tree mortality, disturbance ecology, Scolytinae

## Abstract

The mountain pine beetle (MPB) (*Dendroctonus ponderosae*) is a bark beetle that attacks and kills ponderosa pine (*Pinus ponderosa*), among other pine species throughout the western conifer forests of the United States and Canada, particularly in dense stands comprising large trees. There is information on the stand conditions that the insect prefers. However, there is a paucity of information on how small-scale variation in stand conditions influences the distribution of tree mortality within a stand. I examined the small-scale distribution of ponderosa pine basal area pre- and post a mountain pine beetle infestation, and used geostatistical modeling to relate the spatial distribution of the host to subsequent MPB-caused tree mortality. Results indicated increased mortality in the denser parts of the stand. Previous land management has changed historically open low-elevation ponderosa pine stands with aggregated tree distribution into dense stands that are susceptible to mountain pine beetles and intense fires. Current restoration efforts are aimed at reducing tree density and leaving clumps of trees, which are more similar to historical conditions. The residual clumps, however, may be susceptible to mountain pine beetle populations. Land managers will want to be cognizant of how mountain pine beetles will respond to restoration treatments, so as to prevent and mitigate tree mortality that could negate restoration efforts.

## 1. Introduction

The mountain pine beetle (MPB), *Dendroctonus ponderosae* Hopkins, is a native bark beetle and one of the most important disturbance agents across conifer forests in the western United States. The insect utilizes various species of pines (*Pinus* spp.) as hosts, with ponderosa pine, *Pinus ponderosa* Douglas ex P. Lawson & C. Lawson, being one of the principal hosts [1]. Although an integral component of the ecology of western conifer forests, bark beetle outbreaks can have a significant impact on ecosystem services and processes such as recreation, wildlife habitat, biogeochemistry and snowpack dynamics, among others [2,3,4,5]. Mountain pine beetles also shape forest structure and composition [6]. Mountain pine beetle-caused tree mortality levels range from individual to a few trees killed in small groups under low-level populations to large areas exhibiting extensive tree mortality under eruptive populations.

Group tree mortality caused by MPB is largely the result of host selection and chemical communication. Females select new trees for attack using visual, olfactory and gustatory cues, along with random landings [7,8,9,10]. Chemical communication among MPBs utilizing insect-produced compounds and tree volatiles aggregates hundreds of beetles for attacking a single tree overcoming its defenses. When a tree is fully colonized, an anti-aggregation pheromone arrests further attacks on the tree, causing MPBs to disperse to adjacent trees [11,12]. In lodgepole pine, *Pinus contorta* Douglas ex Loudon, larger diameter trees are attacked first, with surrounding trees being attacked as the larger trees reach their carrying capacity [13]. Although larger trees make up most of the dead ponderosa pines after populations infest a stand, no preference for attacking larger trees first has been demonstrated [14,15].

There is a good understanding of the conditions that make a stand susceptible to MPB, although the plurality of the information addressing ponderosa pine stands’ susceptibility to MPB come from even-aged stands with regular and consistent inter-tree distances from the Black Hills, Washington and Oregon [16,17]. In general, dense stands with larger diameter trees and high host-tree availability are more susceptible to attacks. Little work has been conducted in Colorado or in uneven-aged stands with aggregated clumps of trees separated by open meadows. However, the available data for ponderosa pine in Colorado indicate that stands with a basal area > 17.1 m^2^/ha are more likely to be infested, and more trees > 13 cm in diameter at breast height (dbh), that is 1.4 m above the ground, are attacked [14,15]. For lodgepole pine, the probability of stand infestation in Colorado increases with a basal area > 13.6 m^2^/ha, with trees with a dbh > 18.3 cm [18].

On the other hand, the spatial distribution of forest conditions across a given area and its influence on the spatial distribution of tree mortality has received little attention. A study in the Black Hills of South Dakota and Wyoming, USA indicated that within spatially homogenous stands of ponderosa pine, MPB occurred most frequently in “microcosm stands” with a higher tree density [19]. In Douglas-fir, *Pseudotsuga menziesii* (Mirb.) Franco stands, the Douglas-fir beetle, *Dendroctonus pseudotsugae* Hopkins, caused higher tree mortality in areas of higher basal at a fine scale within larger stands [20]. Aside from these studies, there are no published data on the within-stand spatial distribution of host type or the spatial distribution of bark beetle-caused tree mortality.

The Colorado Front Range (CFR) comprises the eastern slope of the Colorado Rockies, extending from the Colorado–Wyoming border and south of the Arkansas River. There is a history of low-level and periodical eruptive populations of MPB, which can cause varying levels of tree mortality in host trees [21]. In general, the approach to manage bark beetle populations is to mitigate tree mortality in high-value sites, such as campgrounds and ski areas, and across landscapes with vegetation management [22,23]. More recently, management within stands has increased in importance, as it is practiced in the implementation of forest restoration projects.

Historically, in low elevation stands (around 2000 m) prior to about 1860, ponderosa pine forests in the CFR were diverse in structure, with basal areas < 17.1 m^2^/ha, tree diameters up to about 60 cm and aggregated in groups of 2–8 trees [24]. These forest conditions were maintained by frequent fires. Euro-American settlement in the mid-19th century introduced grazing, logging and mining, and during the 20th century, fire suppression. Decades of fire suppression altered low elevation ponderosa pine forests by fostering increases in tree density and continuity across the landscape [24,25,26,27]. These conditions, combined with a changing climate and recent droughts, put these forests at greater risk of MPB infestations and large, stand-replacing fires, which also threaten human lives and structures [28]. In the CFR, large fires have occurred in ponderosa pine forests during the last decade, which burned with uncharacteristically high intensity, creating large areas of complete tree mortality [29]. As a result, forest restoration projects are widely conducted across the CFR, primarily to mitigate fuels and reduce the risk of large fires by returning ponderosa pine forests to more sustainable historical conditions with tree densities, and structures akin to those prior to Euro-American settlement. Approaches vary, but restoration commonly aims to increase stand heterogeneity by increasing variability in tree spacing, reducing tree density, and creating more openings and clumps of trees [28]. Reductions in tree density will help to reduce MPB susceptibility, but maintaining high-density clumps of large diameter trees may also create suitable conditions for MPB infestations [15].

Tools are needed to inform forest health scientists, practitioners and managers on how MPB populations may respond to restoration treatments. This could lead to the implementation of treatments with a lower risk of negating restoration investments by MPB infestations. Understanding how MPB is influenced by the fine-scale spatial variation in stand structure, particularly in uneven-aged stands with aggregated tree distribution, will increase awareness of the interaction between this insect, its hosts and restoration practices. In this study, I examined the spatial distribution of MPB-caused tree mortality in a low-elevation, unmanaged, uneven-aged stand, and how it may relate to the spatial distribution of ponderosa pine basal area across the stand, with the hypothesis that tree mortality occurred primarily in high-density clumps. I concluded that MPB populations caused tree mortality that was spatially concentrated in the denser parts of the stand. This finding can have important implications for the restoration of low-elevation ponderosa pine forests in Colorado.

## 2. Materials and Methods

### 2.1. Study Site

The study was conducted on the Canyon Lakes Ranger District of the Arapaho-Roosevelt National Forest in the northern CFR in August 1997. It is acknowledged that this represents an aged data set. Nevertheless, with the increasingly common implementation of restoration projects in the CFR, the lack of available data on fine-scale susceptibility to MPB, and the effort and cost of conducting these studies, the relevance and utility of the data for informing forest restoration practices is elevated. This is discussed further below.

Low-level infestations of MPB—that is, not an extensive outbreak—occurred in the study area during the early 1990s. Populations had collapsed by the time of sampling, and no new MPB-infested or killed trees were present. The centroid of the area was at about Universal Transverse Mercator (UTM) 13T 449321, 4509610 (WGS84). The site was within the lower Montane Forest Life Zone at an elevation of about 2200 m. The habitat type in the area was *Pinus ponderosa-Pseudotsuga menziesii/Muhlenbergia montana* [30], which is typical of ponderosa pine-dominated stands in the District. Other tree species in the area included limber pine, *Pinus flexilis* James, quaking aspen, *Populus tremuloides* Michx and Rocky Mountain juniper, *Juniperus scopulorum* Sarg. Of these, only limber pine is also a host for MPB. There was no evidence of prior management in the stand, and the study area was relatively flat with no dominant aspect.

### 2.2. Sampling

A 10 m × 10 m grid was superimposed over a 3.1-ha area, and at each grid point, a variable radius plot was established using a basal area factor prism that approximates 4.6 metric BAF (a basal area factor of 20 in US customary units was used during data collection) to determine the basal area at each point. I focused on basal area, as it is a good descriptor of susceptibility to MPB when trees of a suitable size, generally > 15 cm, are present in CFR ponderosa pine [16]. For all plot trees, I recorded species, dbh and tree condition as alive or MPB-killed; no trees appeared to have died due to other causes. Beetle-killed trees were confirmed by the presence of egg galleries under the bark. The pre-MPB (before mortality occurrence) stand basal area was approximated by considering all beetle-killed trees as alive, while the post-MPB (after mortality occurrence) basal area was characterized by including only the residual live trees. As plots were closely spaced, some trees could occur in adjacent plots, but this is common when collecting spatially-referenced data on a small scale.

### 2.3. Data Analysis

To examine the spatial distribution of basal area across the stand—pre- and post-MPB—I used variograms and ordinary kriging. The variogram describes variation between two points at a given distance, and portrays the spatial continuity of the data. The variogram model quantifies the inference that measurements of a variable are more similar to nearby locations, compared to locations separated by larger distances. The squared variation between all points separated by the same distance are plotted against distance. A model similar to a least-square regression is fit to the data points, which can take a variety of functions such as linear, spherical or Gaussian, among others. The variogram can be characterized by its nugget, which represents unexplained error or variation in scales smaller than sampling distances; the range, which is the distance at which the data are no longer spatially correlated; and the sill, which describes the variability of the range. Once the variogram function is calculated, it can be used for kriging, which is an interpolation method used to estimate values at unsampled locations [31,32,33]. Variogram analysis and kriging have been used to conduct spatial analysis in forest entomology and agricultural systems [33,34,35]. I used kriging to interpolate pre- and post-MPB basal areas to build contour maps, which I examined visually.

A Chi-square test was used to compare the distribution of the number of live trees to MPB-killed trees, and the number of live trees pre- and post-MPB across dbh classes. No trees were killed in the dbh classes < 15 cm, therefore these were excluded from the comparison. I examined differences in ponderosa pine basal area and the percentage thereof between pre-MPB and post-MPB conditions by testing the difference of the live tree basal area (pre-MPB - post-MPB) with a Wilcoxon rank sum test [36]. The mean dbh of live ponderosa pine trees was compared with the mean dbh of MPB-killed trees using a Kruskal-Wallis test [36]. The mean dbh of live ponderosa pine pre- and post-MPB were also compared with a Kruskal-Wallis test.

## 3. Results

Ponderosa pine comprised 99% of the trees in the stand. Pre-MPB ponderosa pine basal area and the percentage thereof was significantly higher compared to post-MPB conditions (Table 1). The mean ponderosa pine basal area killed across the stand was 3.6 ± 0.4 m^2^/ha (SE), representing 21.1 ± 1.9 percent of the ponderosa pine basal area.

**Figure 1:**
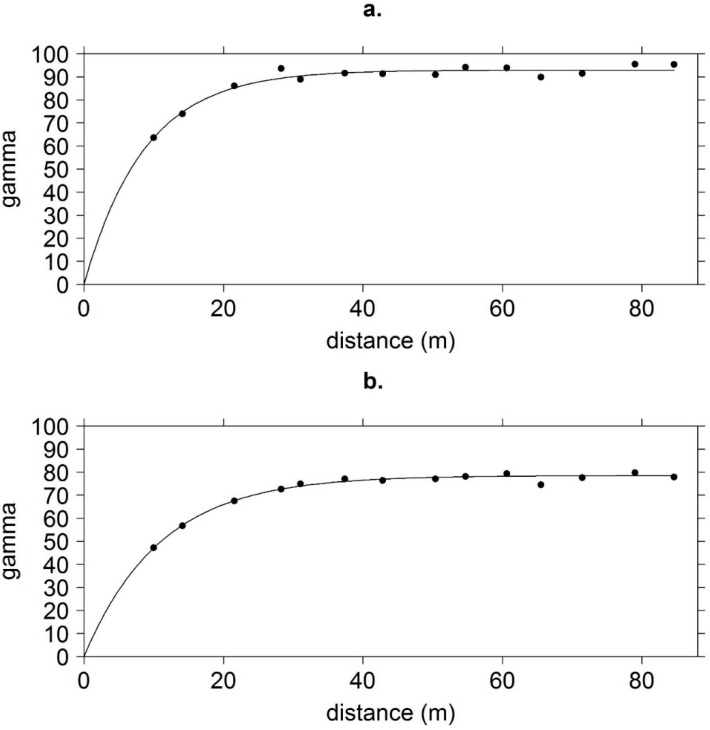
Live ponderosa pine basal area variograms (**a**) pre- and (**b**) post- a MPB event. Gamma is the squared variability of the basal area across distance (m^2^/ha)^2^. Arapaho-Roosevelt National Forest, Colorado, USA.

**Figure 2:**
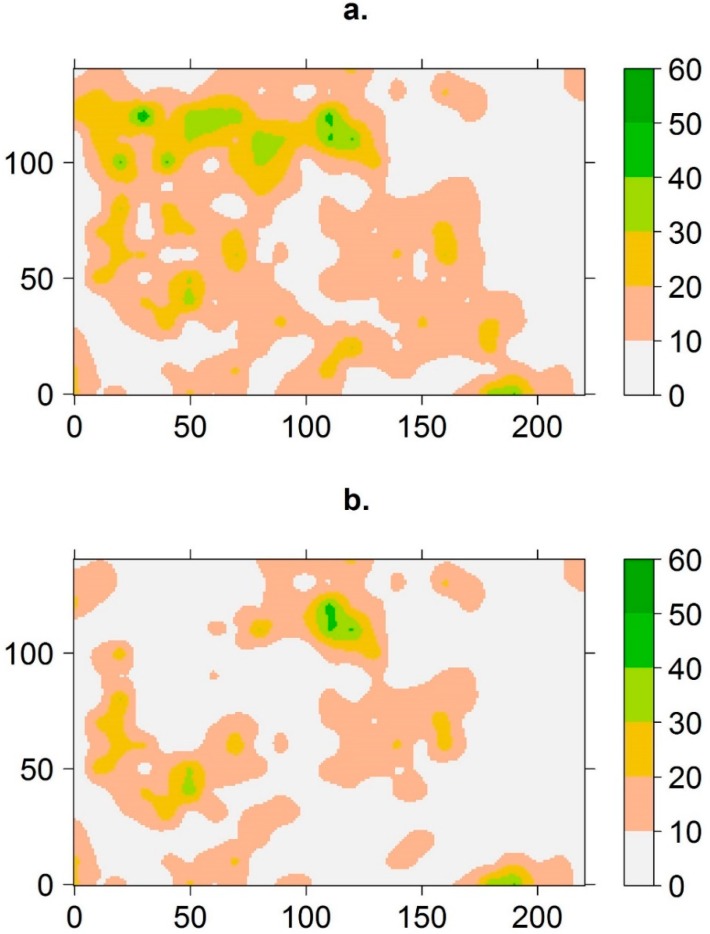
Contour maps from ordinary kriging representing live ponderosa pine basal area (m^2^/ha) (**a**) pre- and (**b**) post- a MPB event; the pre-MPB mean of kriging = 44.1, median = 46.3 and interquartile range = 9.9, and post-MPB mean of kriging = 30.6, median = 32.0 and interquartile range = 7.1. The color scheme adjacent to the maps represents basal area levels, with darker areas representing a higher basal area of live trees. Arapaho-Roosevelt National Forest, Colorado, USA.

I fit spherical models to the variograms of the spatial correlation of live basal area pre- and post-MPB (Figure 1). Visual examination of the contour map of the kriged basal area illustrates where the basal area was reduced by MPB; primarily in the northwestern and southeastern part of the stand, and to a lesser extent in the central portion (Figure 2). These were the denser parts of the stand pre-MPB. The range increased from 9 m pre-MPB to 11 m post-MPB, and the sill of the variogram decreased from 93 m to 79 m, indicating reduced variation in the basal area across the stand. This suggests that MPB-caused mortality resulted in a more homogeneous stand post-MPB.

**Figure 3:**
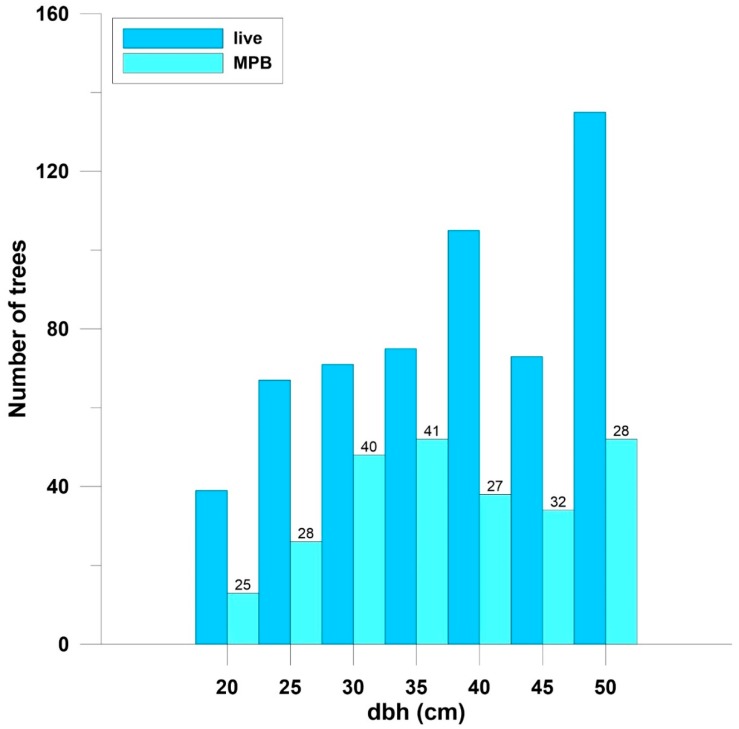
Number of live and MPB-killed ponderosa pine trees across diameter at breast height (dbh) classes. Numbers above MPB bars are the percent of MPB-killed trees per dbh class. The distributions were different; Chi-square = 72.9, df = 10, *p* < 0.0001. Arapaho-Roosevelt National Forest, Colorado, USA.

**Figure 4:**
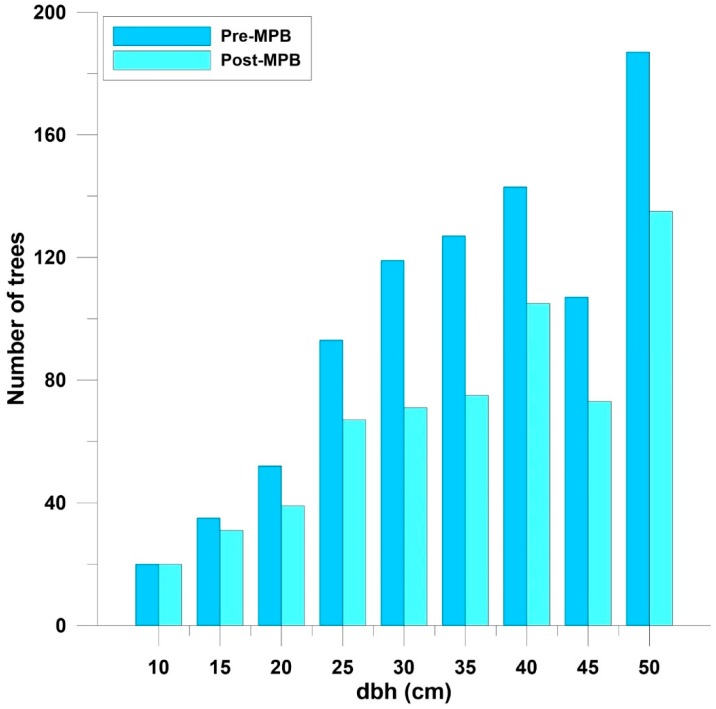
Number of live ponderosa pine trees pre- and post- a MPB event across dbh classes. The distributions were not different; Chi-square = 12.8, df = 10, *p* = 0.24. Arapaho-Roosevelt National Forest, Colorado, USA.

The distributions of the number of live and beetle-killed trees across dbh classes were different (Chi-square = 13.8, df = 6, *p* < 0.03) (Figure 3), primarily due to a higher number of killed trees in the 30 cm and 35 cm classes, and fewer in the 45 cm and 50 cm classes. The distributions of the number of live trees pre- and post-MPB were not different (Chi-square = 5.1, df = 9, *p* > 0.7) (Figure 4), and there were no differences in mean dbh (cm) between MPB-killed trees (38.0 ± 0.8, n = 263) and live trees (38.4 ± 0.5, n = 565) (Chi-square 1.3, df = 1, *p* > 0.3). There were also no differences in mean dbh (cm) between live ponderosa pine both pre-MPB (36.7 ± 0.4, n = 883) and post-MPB (36.3 ± 0.5, n = 616).

## 4. Discussion

Mountain pine beetle-caused tree mortality resulted in a significant reduction of live ponderosa pine basal area, with mortality occurring primarily in the denser parts of the stand. This is consistent with a study in the Black Hills that indicated that MPB-caused tree mortality in ponderosa pine occurred in “microstands” of higher tree density and basal area [19], and with the observed spatial association between Doulas-fir beetle-caused tree mortality and pre-mortality Douglas-fir basal area [20]. Mountain pine beetles also aggregate in lodgepole pine stands, based on stand conditions—namely the location of large-diameter trees [13].

The MPB infestation under which this study was conducted was of low intensity and extent, relatively short-lived, and was limited to ponderosa pine stands. The mean pre-MPB ponderosa pine basal area was 11.8 (0.5) m^2^/ha (Table 1), which is below susceptibility levels. I observed only 21% of the ponderosa pine basal area killed by MPB in the study area. This relatively low mortality level contrasts with a CFR study that reported a high mortality level of 78% in the basal area, but with a much higher pre-MPB basal area of 28 m^2^/ha [14].

A higher percentage of trees was killed in the 22.5 cm or larger dbh classes; this is consistent with other studies in ponderosa pine in Colorado that have reported MPB tree diameter preferences [14,15]. The lack of difference between the dbh of MPB-killed trees as compared to unattacked live trees and the absence of changes observed in the distribution of surviving live trees when compared to pre-MPB live trees are also indicative of the relatively low-level mortality observed. A higher intensity infestation may have resulted in a larger mean diameter of killed trees compared to live trees, as well as a change in the distribution of surviving trees across diameter classes. In addition, a greater change in the range and sill may occur, with higher mortality portraying a more homogenous stand post-MPB.

Restoration treatments in the CFR aim at leaving clumps of trees, which is more consistent with historical conditions [24,28]. A relevant but difficult concept for describing increased tree mortality in the denser areas of a stand is defining what a “clump” of trees is. The probability of infestation by MPB in ponderosa pine in the CFR increases when the ponderosa pine basal area > 17.1 m^2^/ha, and at the individual tree level when dbh >18.2 cm [15]. Therefore, a clump of susceptible trees could be defined as an area where ponderosa pine basal area is > 17.1 m^2^/ha, and comprised mostly of trees > 18.2 cm. Using this as a description of susceptible areas, the plurality of our study area could be considered of low to moderate susceptibility, as visual examination of the pre-MPB contour map of the basal area showed that most of the area had spatially-referenced values < 20 m^2^/ha, yet increased mortality was still higher in the denser areas.

### Context of the Study

Since the data presented in this study was collected years ago (1997), it is important to articulate why it is still relevant and how it fits under current conditions. First, the paucity of information on the influence of small-scale variability of stand characteristics on the distribution of tree mortality can hinder the development of better guidelines for reducing susceptibility to MPB while implementing restoration projects. Second, the examination of small-scale variations in stand conditions is laborious and resource-demanding, limiting the ability to examine large replicated areas. In this study, I examined a 3.1-ha stand. The study by Olsen et al. (1996) [19] comprised an area of 0.9 ha, while the study by Mitchell and Preisler (1991) [13] encompassed only a 0.5-ha stand. Only Negrón et al. (2001) [20] examined larger replicated areas ranging from 4 to 16 ha. Still, all studies confirm the preference of MPB and Douglas-fir beetles for higher-density parts of the stands, and for MPB around large-diameter lodgepole pine. I acknowledge that the sampling area in this study was not extensive, and results in some limitations. For example, an examination of a larger area would likely encompass a wider range of tree density and diameter classes. Restoration projects comprise larger areas than those examined in this study, which challenge the transference of results from small areas to larger stands. These are factors that need to be considered in future studies, in order to offer more comprehensive recommendations to land managers.

A recent large MPB outbreak affected about 1.4 million ha of lodgepole pine forests in Colorado between 2000 and 2013 [37]. However, low-level mortality caused by MPB continues to be part of the biotic disturbance processes in Colorado pine forests [38]. The role of endemic and low-level populations of bark beetles will continue to shape and influence unmanaged and managed stands, and their study remains a priority. Bark beetle outbreaks and other disturbances such as fires, blowdowns and certain forest pathogens are anticipated to increase in frequency and intensity under climate change [39,40,41,42], although many gaps in knowledge and modeling techniques remain to support more accurate assessments [43]. In 2014 and 2015, I conducted walk-through surveys of the study area and saw no additional mortality caused by the recent outbreak, likely because the high-density clumps had already been killed. The recent large outbreak is not unique, as similar events have occurred in the past [44]. In recent years, the research emphasis on bark beetle ecology has shifted to interactions with climate change, but studying the role of low-level populations is still important and supports current management efforts, and will serve to compare how these may change as the climate changes.

With the recent emphasis on restoration efforts in the CFR, the data are relevant and shed light on contemporary management emphasis. Mountain pine beetles’ preference for clumps of high host concentration and associated mortality can have implications for ponderosa pine restoration efforts in the CFR. Many restoration strategies aim for the preservation of large-diameter trees in groups [28]. Based on the results and using the above as a description of clumps, restoration strategies that retain these structures have the potential to increase MPB-caused mortality in residual denser clumps of trees when MPB populations increase. Managers should closely monitor MPB populations near restoration projects prior to and after restoration activities, and if appropriate, implement MPB population mitigation practices such as the timely removal of infested trees, among others. These measures, however, are only suitable for low-level populations and at small spatial scales, and will not be effective under large epidemic conditions [22,45].

## 5. Conclusions

The restoration of degraded forest ecosystems is a crucial endeavor for the conservation of biological diversity, protection of natural resources, human life and property, and maintaining resilient ecosystems, especially in the face of a changing climate. Stand density reduction is considered to be the most effective way to mitigate MPB-caused mortality in ponderosa pine forests [17,23]. The implementation of restoration efforts, however, is costly and requires extensive resources, so managers must consider the unintended effects of restoration efforts. Leaving higher density clumps of large-diameter trees in ponderosa pine restoration is part of creating conditions more similar to those before Euro-American settlement, and that are likely more resilient to disturbance. Findings from this study can help inform land managers and raise the awareness of how MPB activity could impact treated stands, and practice the mitigation of MPB populations, if needed and appropriate, to prevent negating investments in restoration efforts.

## Figures and Tables

**Table 1:** Mean (SE) *Pinus ponderosa* basal area (m^2^/ha) and the percentage of pre- and post-*Dendroctonus ponderosae* (MPB)-caused mortality and their differences (pre-MPB—post-MPB). Differences tested with a Wilcoxon rank sum test, *p* < 0.05. Arapaho-Roosevelt National Forest, Colorado, USA.

Variable	Pre-MPB	Post-MPB	Difference	*p*
Ponderosa pine basal area	11.8 (0.5)	8.2 (0.5)	3.6 (0.4)	<0.0001
Percent Ponderosa pine basal area	77.4 (2.2)	56.2 (2.4)	21.1 (1.9)	<0.0001

## Data Availability

All data are archived at the USDA Forest Service, Rocky Mountain Research Station and are available as: Negron, Jose F. Within-stand spatial distribution of ponderosa pine mortality caused by the mountain pine beetle in the Colorado Front Range. Fort Collins, CO: Forest Service Research Data Archive. https://doi.org/10.2737/RDS-2020-0006.
